# Pencils of Tannin in Affections of the Uterus

**Published:** 1860-04

**Authors:** 


*Pencils of Tannin in Affections of the Uterus.*—This form of application, pointed out by Dr. Becquerel, seems likely to be of service in the treatment of lesions affecting the cavities of the neck and body of the uterus. In particular, in the fungous conditions of their mucous membranes, with consecutive hæmorrhages, the tannin pencils might be advantageously substituted for the intra-uterine injections, which are not always free from danger. Dr. Becquerel's formula is: ℞.—Tannin, 4 parts; gum tragacanth, 1 part; bread-crumb, q. s. to give the proper consistence. These pencils are 5 millimetres in diameter, and 3 centimetres long. To use them, the neck of the uterus is exposed by means of the speculum; a pencil of tannin is introduced by means of the forceps into the os tincæ, and is then pushed into the uterine cavity, and secured there by means of a plug of lint soaked with concentrated solution of tannin. Once in position, the pencil softens and dissolves, and modifies the tissues with which it is in contact. At the end of twelve hours, the plug of lint is withdrawn by means of a thread attached to it. Every three or four days a new pencil is introduced in the same manner; and after a month of this treatment, the fungous state of the mucous membrane progressively disappears, and the hæmorrhages are arrested.—*Bull. Gén. de Téhrap.* and *Edin. Med. Jour.*

